# Supplementary material for: Spatiotemporal dynamics in airborne fungi and allergenic fungal taxa across the United States
Source: Appl Environ Microbiol. 2026 May 21;92(6):e00059-26. doi: 10.1128/aem.00059-26 (PMC13274342; doi:10.1128/aem.00059-26)
Supplement: Supplemental material — Tables S1 to S3; Fig. S1 to S3. [file aem.00059-26-s0001.docx]

Supporting Information for

Spatiotemporal dynamics in airborne fungi and allergenic fungal taxa across the United States

| **Supplementary Table 1**. Site summary details for all 7 sampling locations | | | | | | |
| --- | --- | --- | --- | --- | --- | --- |
| Site | Latitude | Longitude | Elevation (m) | State | National Park (NP) | Biome |
| HAVO | 19.4309 | -155.2579 | 1258 | Hawaii | Hawai’i Volcanoes NP | tropical rainforest |
| OLYM | 48.0065 | -122.9727 | 599 | Washington | Olympic NP | Pacific coast and temperate forest |
| ROMO | 40.2783 | -105.5457 | 2760 | Colorado | Rocky Mountain NP | montane |
| VOYA | 48.4132 | -92.8303 | 425 | Minnesota | Voyageurs NP | boreal forest and temperate deciduous forest |
| GRSM | 35.6334 | -83.9416 | 810 | Tennessee | Great Smoky Mountains NP | temperate deciduous forest |
| EVER | 25.3910 | -80.6806 | 1.25 | Florida | Everglades NP | subtropical wetlands |
| ACAD | 44.3800 | -68.2600 | 157 | Maine | Acadia NP | Atlantic coast, boreal and deciduous forest |

**Supplementary Fig. 1:** Fungal richness, measured as the number of observed taxa, over time across all 7 sites. Panels (A-G) show temporal patterns per site and panel (H) includes the median overall fungal richness by site. **Supplementary Fig. 2:** Bray-Curtis dissimilarity comparing all samples to the baseline (average of the oldest ten samples sequenced, shown in red) at each site to assess storage impact on archived filters. If storage time systematically altered community composition, we would expect dissimilarity to increase linearly over time as samples spent progressively less time in storage. Instead, dissimilarity patterns follow seasonal cycles at all sites, with values ranging from approximately 0.4 to 1.0 throughout the year. Samples at HAVO and EVER, contain the most complete annual sequencing coverage, demonstrate seasonal cycling rather than storage degradation. At ROMO and VOYA, early-year samples were not retained or sparse, so baseline samples were averaged during late spring/early summer with the linear increase in dissimilarity for later samples reflecting seasonal differences rather than storage artifacts and are shown here for transparency.

**Supplementary Fig. 3:** Total allergenic concentrations for each sampling day across sites reported as fungal genome equivalents m^-3^ of air. Panels (A-G) are labeled by the site from left to right: Hawai'i Volcanoes National Park, HI (HAVO), Olympic National Park, WA (OLYM), Rocky Mountain National Park, CO (ROMO), Voyageurs National Park, MN (VOYA), Great Smoky Mountains National Park, TN (GRSM), Everglades National Park, FL (EVER), and Acadia National Park, ME (ACAD). Each stacked bar represents an individual sampling date, showing the DNA concentrations of allergenic fungal genera. Colors represent different allergenic genera as shown in the legend.

| **Supplementary Table 2**. List of ASVs shared across all sites. | | | | | |
| --- | --- | --- | --- | --- | --- |
| Phylum | Class | Order | Family | *Genus* | Species |
| Ascomycota | Pezizomycetes | Pezizales | Pyronemataceae | *Pyronema* | ASV_1 |
| Ascomycota | Dothideomycetes | Capnodiales | Cladosporiaceae | *Cladosporium* | ASV_2 |
| Basidiomycota | Agaricomycetes | Polyporales | Fomitopsidaceae | Fomitopsidaceae | ASV_4 |
| Ascomycota | Dothideomycetes | Pleosporales | Pleosporaceae | *Alternaria* | ASV_5 |
| Basidiomycota | Agaricomycetes | Polyporales | Polyporaceae | *Trametes* | ASV_10 |
| Ascomycota | Eurotiomycetes | Eurotiales | Aspergillaceae | *Aspergillus* | ASV_11 |
| Ascomycota | Pezizomycetes | Pezizales | Pyronemataceae | *Heydenia* | ASV_14 |
| Ascomycota | Dothideomycetes | Capnodiales | Cladosporiaceae | *Cladosporium* | ASV_15 |
| Basidiomycota | Agaricomycetes | Polyporales | Meruliaceae | *Scopuloides* | ASV_17 |
| Basidiomycota | Agaricomycetes | Hymenochaetales | Rickenellaceae | *Resinicium* | ASV_30 |
| Ascomycota | Eurotiomycetes | Eurotiales | Aspergillaceae | *Aspergillus* | ASV_39 |
| Basidiomycota | Agaricomycetes | Russulales | Stereaceae | *Stereum* | ASV_43 |
| Basidiomycota | Agaricomycetes | Agaricales | Schizophyllaceae | *Schizophyllum* | ASV_49 |
| Ascomycota | Sordariomycetes | Trichosphaeriales | Trichosphaeriaceae | *Nigrospora* | ASV_55 |
| Basidiomycota | Tremellomycetes | Filobasidiales | Filobasidiaceae | *Filobasidium* | ASV_56 |
| Ascomycota | Eurotiomycetes | Eurotiales | Aspergillaceae | *Aspergillus* | ASV_200 |
| Ascomycota | Dothideomycetes | Pleosporales | Sporormiaceae | *Preussia* | ASV_229 |
| Basidiomycota | Agaricomycetes | Polyporales | NA | NA | ASV_256 |
| Ascomycota | Dothideomycetes | Pleosporales | Didymellaceae | *Epicoccum* | ASV_420 |
| Ascomycota | Eurotiomycetes | Eurotiales | Aspergillaceae | *Aspergillus* | ASV_692 |

| **Supplementary Table 3**. Complete list of explanatory variables tested, including weather variables, atmospheric chemistry measurements, and environmental variables associated with each sample. | | | |
| --- | --- | --- | --- |
| Weather Variables | Atmospheric Chemistry | Environmental Variables |  |
| Air Temperature | PM_2.5_ | Solar radiation |  |
| Minimum Temperature | PM_10_ | Evapotranspiration |  |
| Maximum Temperature | SO_4_ | Surface soil moisture |  |
| Relative Humidity | Na |  |  |
| Precipitation | K |  |  |
| Wind direction | K |  |  |
| Wind speed | NO_3_ |  |  |
| Wind gusts | OC |  |  |
|  | S |  |  |
|  | Dust Composite metric* |  |  |

*Dust composite metric includes aluminum (Al), silicon (Si), calcium (Ca), iron (Fe), and titanium (Ti).
